# Calculating age-adjusted cancer survival estimates when age-specific data are sparse: an empirical evaluation of various methods

**DOI:** 10.1038/sj.bjc.6602976

**Published:** 2006-01-24

**Authors:** A Gondos, D M Parkin, E Chokunonga, H Brenner

**Affiliations:** 1 Department of Epidemiology, German Centre for Research on Ageing, Bergheimer Str. 20, Heidelberg 69115, Germany; 2 Unit of Descriptive Epidemiology, International Agency for Research on Cancer, Lyon, France; 3 Clinical Trial Service Unit and Epidemiological Studies Unit, Nuffield Department of Clinical Medicine, University of Oxford, Oxford, UK; 4 Zimbabwe National Cancer Registry, Harare, Zimbabwe

**Keywords:** cancer survival, age-adjusted survival, sparse data

## Abstract

We evaluated empirically the performance of various methods of calculating age-adjusted survival estimates when age-specific data are sparse. We have illustrated that a recently proposed alternative method of age adjustment involving the use of balanced age groups or age truncation may be useful for enhancing calculability and reliability of adjusted survival estimates.

Age adjustment is widely used in studies comparing survival of different cancer patient populations. Most commonly, the comparison is made by calculating a weighted average of age-specific survival estimates, using weights reflecting the age distribution of some defined standard population. There has, however, been a large diversity in the practice of age adjustment, mainly concerning the definition, the number and width of the age groups, and methods to overcome difficulties when the age-specific survival could not be calculated. Such situations are not uncommon in comparative survival studies, particularly when data for rare cancers or data from registries covering smaller populations is involved. Examples of the practice of age adjustment of cancer survival are given in Table 1.

Recently, an alternative method was proposed for age adjustment of survival estimates (Brenner *et al*, 2004), in which weights are assigned to the patients before one carries out the survival analysis. Weights are determined as the percentage of patients in the age group the patient belongs to in the standard population divided by the corresponding percentage in the study population. Survival analysis is carried out using the weighted individual data, without the need to calculate age-specific survival estimates (Brenner *et al*, 2004).

In this paper, we empirically evaluate and compare the performance of different options of age adjustment methods in situations when age-specific data are sparse.

## MATERIALS AND METHODS

### Data set

Data from the Zimbabwe National Cancer Registry (ZNCR) (Chokunonga *et al*, 2000; Parkin *et al*, 2003) were used. The registry, established in 1985, covers the population of the Zimbabwean capital, Harare. In terms of operational circumstances, which have been described in detail elsewhere, the registry may be considered as a typical example for an urban developing country cancer registry with appropriate data quality outcomes (Sankaranarayanan *et al*, 1998; Parkin *et al*, 2003; Chokunonga *et al*, 2004). Survival results for a large number of cancer sites were recently published elsewhere (Gondos *et al*, 2004).

We assessed various options of age adjustment of 5-year survival estimates among patients diagnosed with five different types of cancers in 1993–1997 and followed up until 31 December 1999: skin melanomas, breast, cervical and prostate cancer and lymphomas. Breast and cervical cancers were included because they were represented by relatively large samples. Prostate cancer, for which only 3-year survival could be calculated due to the lack of patients with a 5-year follow-up time, was included because of the unusual age distribution (high proportions of older patients). Lymphomas were included because of the uniquely wide age range of the patients, and skin melanomas were selected as an example of an analysis with a very small sample.

### Calculation of age-adjusted survival

The site-specific World Standard Cancer Patient Populations (WSCPP) were used as standard populations (Black and Bashir, 1998). Adjustment of the survival estimates was carried out according to the traditional method, and the alternative method recently proposed by Brenner *et al* (2004), using the age categorisation schemes described below. Throughout this paper, relative rather than absolute survival estimates are presented. The relative survival estimates were calculated according to Hakulinen's method (Hakulinen, 1982), using the WHO life tables for Zimbabwe (WHO, 2001). The calculations were carried out using the SAS macros *periodh* (Brenner *et al*, 2002) and *adperiodh* (Brenner *et al*, 2004).

### Scheme 1: Age adjustment with fixed age group width

First, for each cancer site, we classified the patients by 5-, 10-, 15- and 30-year age groups. With each of these classifications, both the youngest and the oldest age groups were selected so that they actually included patients, and the age of the youngest/oldest patient determined the first/last age group (eg, if the youngest patient was 28, the first 5/10/15/30-year age group was 25–29/20–29/15–29/0–29, respectively).

### Scheme 2: Collapsing the youngest and oldest age groups

If needed, that is, if the age adjustment failed, we applied modifications to the boundaries of the youngest and the oldest age groups to enhance calculability: the boundaries of these age groups were modified so that the age-specific survival in these (youngest and oldest) age groups could be calculated. Age groups in between were left unchanged, except for the 30-year age groups, where the shifting of the first or last age group affected the middle age group as well.

### Scheme 3: Balanced age groups

Here, we reorganised the age groups in such a way that the number of observations in the age groups would be approximately evenly distributed. The number of age groups was varied between 3 and 5. The boundaries of these ‘balanced’ age groups were aligned to the nearest of those of the original 5-year age groups.

### Calculation of truncated survival

Age-specific survival estimates are often unreliable for older age groups in data from developing countries. Often, as is the case in our study, the standard population gives more weight to the oldest age group than does the study population. In these cases, the adjusted survival estimate can easily become unreliable, as the adjustment assigns a large weight to an unreliable age-specific survival estimate. We therefore repeated all calculations with a truncated age range (0–74 years), following the practice in the so far largest comparative survival study from developing countries (Sankaranarayanan *et al*, 1998).

## RESULTS

Table 2 shows the numbers of patients by age group in the Zimbabwean cancer populations, illustrates the differences between the age distributions of the study and the standard populations, and indicates the age groups for which the 5-year age-specific survival estimate could not be calculated. The WSCPP include a much higher proportion of patients in the oldest 2–4 age groups than the Zimbabwean patient populations.

Table 3a provides survival estimates adjusted by the traditional and the alternative method, with all ages included, according to the different schemes we applied. With the traditional method, the fixed age group classifications often failed, due to a failure in calculating 5-year age-specific survival estimates. With collapsed or balanced age groups, the traditional age adjustment became feasible in most cases. The alternative method was feasible even with most fixed age group categorisations, except for the 5-year categories for skin melanomas and lymphomas, where one age group was empty and therefore the weight to be assigned to the patients in the age group could not be calculated. With both the traditional and the alternative method, the application of different age groups resulted in different adjusted survival estimates for all cancer types studied. With the use of balanced age groups, variation was strongly reduced.

Table 3b summarises and compares the results obtained by calculating adjusted survival estimates with all ages involved and with truncated cancer patient populations. The truncation did not alter the crude survival estimates significantly: the differences between the crude and the truncated crude survival estimates were between 0.6 and 3.6% units. However, the variation in the adjusted survival estimates among the different categorisation schemes was strongly reduced for all cancer sites.

## DISCUSSION

With the traditional method, the calculation of 5-year age-specific survival estimates often failed in age groups with a few patients only. Failures could mostly be overcome by the application of different age categorisation schemes, that is, by collapsing or balancing the age groups. When using the alternative method (Brenner *et al*, 2004), calculability was generally very good, even with the fixed age group categorisation schemes.

However, with both methods, the different age group classifications produced age-adjusted estimates with a rather large variability, mainly because of the assignment of large weights for the older age groups in which data were sparse in the ZNCR. These variations could be effectively reduced using balanced age groups and by restricting the analysis to a truncated age range up to 74 years.

There is no theoretically best practice with regard to the number of age groups, their width and the boundaries of the individual age group classifications. For practical purposes, however, adjusted estimates should be reasonably consistent, no matter what age classifications are used. Limitations in data quality frequently impair the reliability of age-specific data among older patients, particularly in case of patient populations from developing countries (Sankaranarayanan *et al*, 1998). In such cases, the calculation of truncated adjusted survival may provide estimates of improved reliability and comparability. On the other hand, truncation means that the survival experience of older patients is neglected, which would be justified only if the proportion of these patients is very small. The use of balanced age groups is not affected by this limitation and may be preferred if the exclusion of older patients is of concern.

In looking at the results, the following limitations should be kept in mind. Our empirical evaluation is based on five cancer sites from one cancer registry only. The cancer sites were chosen to represent various sample sizes, age distributions, and higher and lower survival patterns, and therefore reflect a variety of scenarios encountered in comparative analyses of survival. While the problems of sparseness of data and discrepancy between the age distribution of the study population and the standard population were probably more extreme than in most other practical applications, such extreme data situations may facilitate the demonstration of the implications of various analysis strategies under the above conditions. We did not include standard errors of survival estimates obtained with the various methods. As recently demonstrated elsewhere (Brenner and Hakulinen, 2005), the alternative method often provides estimates with a smaller standard error.

In summary, our results on the one hand illustrate that the enhanced calculability of age-adjusted survival estimates by the alternative method may be relevant in practice. Nevertheless, the unreliability of estimates in case of sparse data within age groups may remain a concern for both the traditional and the alternative method. In such situations, the use of balanced age groups or the calculation of truncated age-adjusted survival estimates may be useful analytical options.

## Figures and Tables

**Table 1:** Examples of age adjustment of population-based cancer survival estimates in the recent literature

**Authors (year)**	**Study/registry**	**Cancer sites**	**Age groups (years)**	**Standard population**	**Note**
Aareleid *et al* (1998)	EUROCARE-2	Testicular cancer	15–44, 45–54, 55–64, 65–74, 75+	All cases with malignant testicular cancer included in the study, in addition time-series comparison using EUROCARE-1	Age adjustment often failed
Sant *et al* (1998)	EUROCARE-2	Malignant brain tumours	15–44, 45–54, 55–64, 65–99	All cases with malignant brain tumours included in the study	Age adjustment often failed
Sant *et al* (2001)	EUROCARE-2	10+ different sites	15–44, 45–54, 55–64, 65–99	All cases included in the study, by site	
Capocaccia *et al* (2003)	EUROCARE-3	10+ different sites	15–44, 45–54, 55–64, 65–74, 75+	All cases included in the study, by site	Missing age-specific values modelled
Dickman *et al* (1999)	Finland	10+ different sites	0–14, 15–29, 30–44, 45–59, 60–74, 75+	Cancer patient populations of the most recent period in the study (1985–1994)	
Sankaranarayanan *et al* (1998)	IARC: Cancer survival in developing countries	10+ different sites	0–34, 35–44, 45–54, 55–64, 65–74, 75+	World Standard Cancer Patient Populations	Age groups were collapsed when age-specific survival could not be calculated, additional analyses for truncated age range 0–74 years
Wang *et al* (2003)	Singapore	Cervical cancer	0–39, 40–49, 50–59, 60–69, 70+	World Standard Cancer Patient Populations	
Chokunonga *et al* (2004)	ZNCR	Cervical Cancer	0–34, 35–44, 45–54, 55–64, 65–74, 75+	World Standard Cancer Patient Populations	

IARC=International Agency for Research on Cancer, Lyon, France; ZNCR=Zimbabwe National Cancer Registry, Harare.

**Table 2:** Age distributions of cancer patient populations in Zimbabwe and of the WSCCP, and the calculability of age specific 5-year survival estimates, by 5-year age groups

	**Skin melanoma**				**Breast**				**Cervix**				**Prostate**				**Lymphomas**			
	**Zimbabwe**			**WSCCP**	**Zimbabwe**			**WSCCP**	**Zimbabwe**			**WSCCP**	**Zimbabwe**			**WSCCP**	**Zimbabwe**			**WSCCP**
**Age group (years)**	***N***	**%**	***F* a**	**%**	***N***	**%**	***F* a**	**%**	***N***	**%**	***F* a**	**%**	***N***	**%**	***F* a**	**%**	***N***	**%**	***F* a**	**%**
0–4	—	0		0	—	0		0	—	0		0	—	0		0	4	3.4		2.8
5–9	—	0		0.1	—	0		0	—	0		0	—	0		0	5	4.2	†	2.7
10–14	—	0		0.4	—	0		0.1	—	0		0.1	—	0		0	6	5.1		2.7
15–19	—	0		0.9	—	0		0.3	—	0		0.3	—	0		0	5	4.2	†	2.8
20–24	—	0		1.8	5	4.4	†	0.9	1	0.4	†	1.1	—	0		0	5	4.2		3.1
25–29	2	4.9	†	3.3	2	1.8		2.1	14	6.0		2.7	—	0		0	13	11.0		3.5
30–34	3	7.3		4.9	5	4.4		4	26	11.2		5.2	—	0		0.1	17	14.4		4
35–39	2	4.9		6.6	20	17.5		6.4	27	11.6		8.2	—	0		0.2	18	15.3		4.6
40–44	2	4.9		7.8	13	11.4		8.5	32	13.8		10.8	—	0		0.3	16	13.6		5.4
45–49	7	17.1		8.7	20	17.5		10	36	15.5		12.1	—	0		0.8	11	9.3		6.2
50–54	4	9.8		9	13	11.4		10.5	24	10.3		12	2	5.7		1.6	7	5.9	†	7
55–59	6	14.6		8.9	11	9.7		10.2	24	10.3		10.9	2	5.7		3.3	6	5.1		7.7
60–64	6	14.6		9.1	14	12.3		9.7	21	9.1		9.8	8	22.9		6.2	3	2.5	†	8.5
65–69	6	14.6		8.7	3	2.6		9	12	5.2	†	8	6	17.1		8.7	0	0		8.4
70–74	0	0.0		8.3	2	1.8		8.2	9	3.9		6.5	7	20.0		12.1	1	0.9		8.2
75–79	2	4.9		7.8	6	5.3		7.5	4	1.7	†	5.2	6	17.1		16.4	0	0		7.9
80–84	1	2.4		7.2	—	0		6.7	1	0.4		4.1	1	2.9		21.8	0	0		7.5
85+	—	0		6.5	—	0		5.9	1	0.4	†	3	3	8.6		28.5	1	0.9	†	7
																				
Total	41				114				232				35				118			

WSCCP=World Standard Cancer Patient Population.

a F(ailure): Crosses (†) indicate that the age-specific survival estimate for the 5th year could not be calculated in an age group that was otherwise not empty.

**Table 3:** (a) The 5-year relative survival (in %) of Zimbabwean cancer patients, adjusted to WSCPP and (b) summary of calculating truncated survival estimates: differences in the crude estimates, and ranges of the adjusted survival estimates for all ages and truncated cancer patient populations

	**Skin melanoma**		**Breast**		**Cervix**		**Prostate (3 years survival)**		**Lymphomas**	
*(a) 5 year relative survival (in %) of Zimbabwean cancer patients, adjusted to WSCPP*										
Crude relative survival	49.9		37.9		30.5		27.1		23.1	
Age-adjusted survival	Traditional method	Alternative method	Traditional method	Alternative method	Traditional method	Alternative method	Traditional method	Alternative method	Traditional method	Alternative method
										
*Fixed age group width (years)*										
5	—*	—	—	48.1	—	32.5	46.3	45.6	—	—
10	—	40.9	54.1	46.2	—	31.2	39.6	33.8	—	37.5
15	—	44.6	51.3	43.1	—	31.2	21.1	21.5	—	48.6
30	—	49.2	38.3	38.9	30.0	29.1	26.8	26.9	—	39.9
										
*Collapsed age group width (years)*										
5	—	44.9	56.6	48.1^a^	34.0	32.5^a^	46.3^a^	45.6^a^	—	38.0
10	37.3	40.9^a^	54.1^a^	46.2^a^	36.0	31.2^a^	39.6^a^	33.8^a^	18.2	37.5^a^
15	41.5	44.6^a^	51.3^a^	43.1^a^	29.9	31.2^a^	21.1^a^	21.5^a^	23.9	48.6^a^
30	49.3	49.2^a^	38.3^a^	38.9^a^	30.0^a^	29.1^a^	26.8^a^	26.9^a^	23.7	39.9^a^
										
*Balanced age groups*										
5 age groups	41.6	43.0	39.1	39.2	29.3	28.7	21.3	21.9	—	28.3
4 age groups	41.4	43.1	38.7	39.0	29.9	29.5	21.3	21.8	24.6	24.6
3 age groups	48.0	48.8	36.1	35.4	27.6	28.7	21.4	21.7	17.7	17.9

**Summary**	**Skin melanoma**		**Breast**		**Cervix**		**Prostate (3 years survival)**		**Lymphomas**	
*(b) Summary of calculating truncated survival estimates: differences in the crude estimates, and ranges of the adjusted survival estimates for all ages and truncated cancer patient populations*										
Difference between all age and truncated crude relative survival	−2.6		2.2		0.6		−3.6		0.6	
Range (highest–lowest) of adjusted survival estimates	Traditional method	Alternative method	Traditional method	Alternative method	Traditional method	Alternative method	Traditional method	Alternative method	Traditional method	Alternative method
All ages	12.0	8.2	20.5	12.7	8.4	3.8	25.2	24.1	6.9	30.7
Truncated	4.2	4.5	6.6	4.8	4.5	1.9	0.9	1.5	5.6	12.7

WSCCP=World Standard Cancer Patient Population.

a Values are the same as in the fixed age group analysis, as there was no need to collapse age groups.

* Age specific survival could not be calculated.
